# Encounters with fierce dogs and itchy bedbugs: why my first field work failed

**DOI:** 10.1186/1746-4269-10-39

**Published:** 2014-05-02

**Authors:** Ingvar Svanberg

**Affiliations:** 1 Uppsala Centre for Russian and Eurasian Studies, Uppsala University, Box 514, SE-751 20 Uppsala, Sweden

**Keywords:** Ethnobiology, Ethnography, Fieldwork, Companion animals, Cultural zoology

## Abstract

This essay, which is the fifth in the series “Recollections, Reflections, and Revelations: Personal Experiences in Ethnobiology”, is a personal reminiscence by the researcher on his first field experience in Turkey in the late 1970s, which was a failure from an ethnobiological point of view but a success for a social scientist pursuing Turkic studies. The author later returned to ethnobiology during subsequent fieldwork on the Faroes.

## Becoming a scholar

I was brought up in Bergslagen―a mining district in the middle of Sweden―in the 1950s and 1960s. From an early age I was interested in animals and plants and had dogs, cats, birds, frogs, snakes and aquarium fish as my companions. As a child I always found pleasure in discussing old folk botanical aspects with my grandmother, who born in the 1890s and brought up under poor circumstances had great knowledge of plants. Although my family was more urban than rural, hunting and fishing remained part of our life. Later, as a high school student, my interest in reptiles and amphibians led me to various parts of the Mediterranean, in particular to the southern Balkan Peninsula, a region of rich herpetofauna and folk cultural life. Collecting lizards, frogs and newts in the field provided plenty of opportunities to encounter ancient folk beliefs about (and prejudices against) these animals. So my interest for studying what we nowadays call ethnobiological perspectives goes way back.

However, there was no such subject as ethnobiology at the university in the early 1970s. Since I was interested in complex relationships between human beings and other organisms I decided that cultural anthropology was my field. I was trained accordingly as an anthropologist, and went on to specialise in European ethnology. My early scholarly interest focused on how ethnic minorities and rural people utilised marginal areas and how they sustained on biological resources in their vicinity. Key words of the time—we are talking mid-1970s here—were ecology, niches and ethnicity, but also traditional plant use, local handicraft, food customs and animal myths. With great interest I read an inspiring book by Kerstin Eidlitz, one of my own professors, about plant and animal utilisation in harsh environments [1]. I was encouraged early on as well by Phebe Fjellström’s 1964 study of the importance of mountain angelica (*Angelica archangelica*) among the Sámi [2], and Israel Ruong’s analysis on how Sámi nomads and settlers were sustained by a livelihood based mainly on trapping and gathering in the 1930s [3]. Nils Storå’s works on local fishing, hunting and waterfowl catching in northern Eurasia was also inspiring to me [4]. I found Lars Sundström’s lectures in African anthropology on fishermen and ecology in the Niger River area stimulating [5]. Theoretical literature of interest available to me was primarily in cultural ecology and peasant studies [6,7]. Wolfram Eberhard’s broad scholarship in historical sociology inspired my way of writing [8].

The closest I came to ethnobiology as an undergraduate was a research paper, based on interviews, on hallucinogenic and psychotropic plants used as substitutes for marijuana or maybe for fun by young ‘drug addicts’ (rather teenagers and drop-outs). I also investigated various kinds of smoking pipes, often with Asian backgrounds, used by these hippie-inspired ‘addicts’. The study of subcultures was very much in vogue at the time. My teachers were amused and approved of my undertakings.

As a graduate, I started with an historical investigation of a hitherto almost unknown group of sedentary Sámi basket makers and horse slaughterers that lived in central Sweden during the eighteenth and nineteenth centuries. I became acquainted with historical methods and source criticism while tracking down this vanished and forgotten culture in archival sources. This was a fascinating study, an investigation into a history previously unheard of, which kept me busy for several years. I built up a picture from scarce and dispersed source material. My focus was on ethnicity and its relationship to ecological aspects of rural economies. Gathering, hunting, crafts and animal husbandry had been important for this now assimilated group of Sámi. They exploited a marginal niche and gathered wild plants for various purposes. Hunting predatory mammals for bounty was still an important source of income for them as late as in the mid-nineteenth century. Their role of slaughtering horses and skinning fur dogs for the peasants living in the same area was of immense importance. Both activities were regarded as taboo for the Swedish farmers, and they had to rely on their Sámi neighbours for these tasks. Especially dog furs were much in demand by the peasants, but they were, due to these taboo rules, not allowed to kill their own dogs. Instead they hired the Sámi [9]. The importance of wild animals, utilisation of wild plants and traditional dog breeding became interesting aspects, and I gathered a lot of comparative records from the sources on these topics.

## In search for a field

However, I was longing for fieldwork with ‘real people’, not just historical individuals in church records and other administrative sources. Therefore I was interested in finding a small, contemporary marginal group that, in the same way as these Sámi once did, received their income by utilising a niche not used by the majority. I was looking for something similar, such as an ethnic community living a rather traditional way of life within the borders of Europe. I looked again towards the south-east, to the Balkans, where as a high school student I caught frogs and salamanders a few years earlier.

So I travelled extensively around the Balkan Peninsula, visiting remote villages in Greek and Yugoslavian Macedonia (but was harassed by police in the Bulgarian part). Since these were all regions of the old Ottoman Empire I also went to Turkey. I met peasants, shepherds and various peripatetic groups, all of which were of great interest. This part of Europe I experienced late in the 1970s, at least in my eyes, a period still very marked by traditional lifestyles. It was also to some extent economically backward. Ottoman structures persevered. It was also a time when anthropology students were expected to do their field work in a local community, preferably a remote village somewhere. Participant observation was the most preferable method recommended. And I found these villages, settled with Sarakatsans, Juruči (Yörük), Turks, Vlachs (Aromâni), Jifti (Roma) and other interesting ethnic groups. I made interviews in the villages about local economy, documented food habits, and recorded various peculiar customs. I still have notes about ways of preparing and using hedgehogs as food and medicine among various ethnic groups in the southern Balkans and Anatolia, and interesting data on the significance of the crow in Balkan tooth-lore, for instance.

After several subsequent visits to Yugoslavia, Greece and western Turkey, I decided to give the Juruči of eastern Macedonia (Yugoslavia) a try. They were a challenge, living in remote villages up high in the mountains. A local veterinarian had promised to help me establish contacts with them.

I cannot say that my professor was excited with my field setting for my PhD project–rather she was sceptical–but the opportunity to study shepherding, dog keeping and sitting postures (!) in the field was deemed acceptable. She became more encouraging when I promised to study enculturation, especially to document how the villagers of nomadic and sedentary background transferred their way of sitting and standing to the children (she had read a book by anthropologists Margaret Mead and Gregory Bateson from Bali about this). She was particularly interested in animal myths and customs related to physiological conditions, and she loved my Balkan and Anatolian records on customs connected with tooth-shedding, with formulas invoking crows, sometimes dogs, stork, and mice [10].

**Figure 1:**
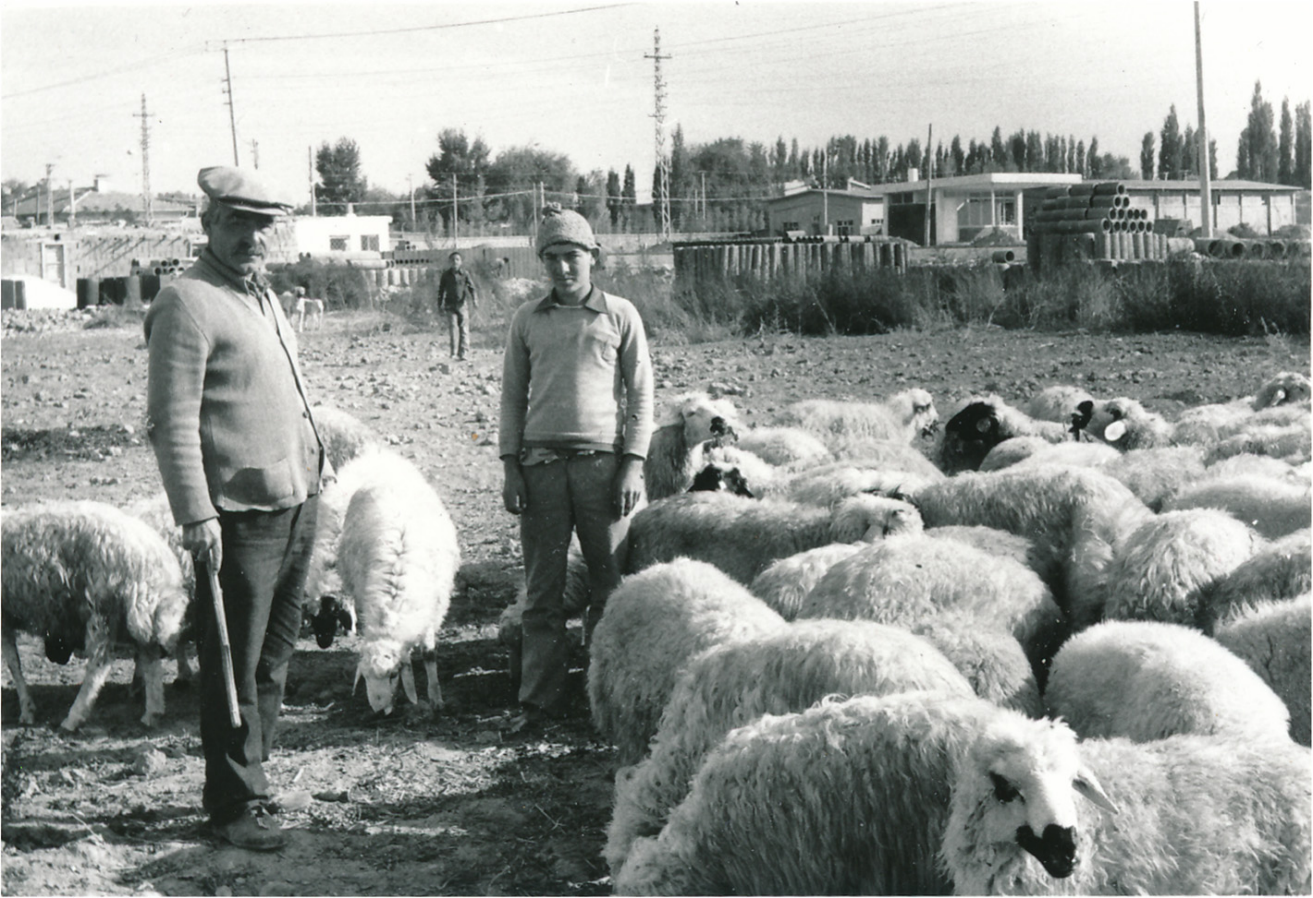
At the Yörük settlement in Niğde (Photo Ingvar Svanberg).

I had met the Juruči in the town of Radovish, but never really established any contact with them. It was usually easy to begin talking to people in the villages and markets, but the Juruči were reticent, shy or uncooperative. Policemen also stopped me when I tried to access their far-off villages in the Plačkovica Mountains. Eventually, with the help of several people I got permission from the Yugoslav Embassy in Stockholm and the authorities in Skopje. I also had some grants for doing Turkic research. I was only waiting for a written permission from a research institute in Skopje, but it never arrived. I tried in vain several times, but never received any answer. This is now a long time ago, before modern computers with internet connections; instead we had to use tricky telephones and unreliable mail, and we had no common language in which to communicate although I provided letters in the Macedonian language. Anyway, I was waiting, and waiting, but no answer arrived. After discussions with colleagues of their experiences in Yugoslavia—foreign field working social scientists were seldom accepted at this time in the country—I eventually decided to use my research money on the probably related Yörüks in Anatolia, whom I had met during two previous trips to the interesting town Niğde (Figure 1).

## Field work in Anatolia

Turkey was not especially open-minded towards foreign scholars either, but it was easy to get around as an outsider and I could stay for several months without permission. I talked to colleagues who had done linguistic and ethnographic field work in Turkey, and I followed their example just going there (some colleagues had actually been expelled even though they had the required permissions). When I returned to Niğde in autumn 1979, my intention was to settle in a neighbourhood (*mahalle*) with Yörük households in the outskirts of the town. It was a family group of Karahacılı Yörüks; all households were related to each other. As far as I remember, there were nine households at the time, sustaining on semi-nomadic shepherding, carpet making, and agriculture (sugar beets). These Yörük households spent the winter season in this neighbourhood, and in the spring many family members moved with their cattle and sheep up to the *yayla*, the summer pastures, in the mountains. They also, like most rural people on the Anatolian countryside, did some plant gathering.

**Figure 2:**
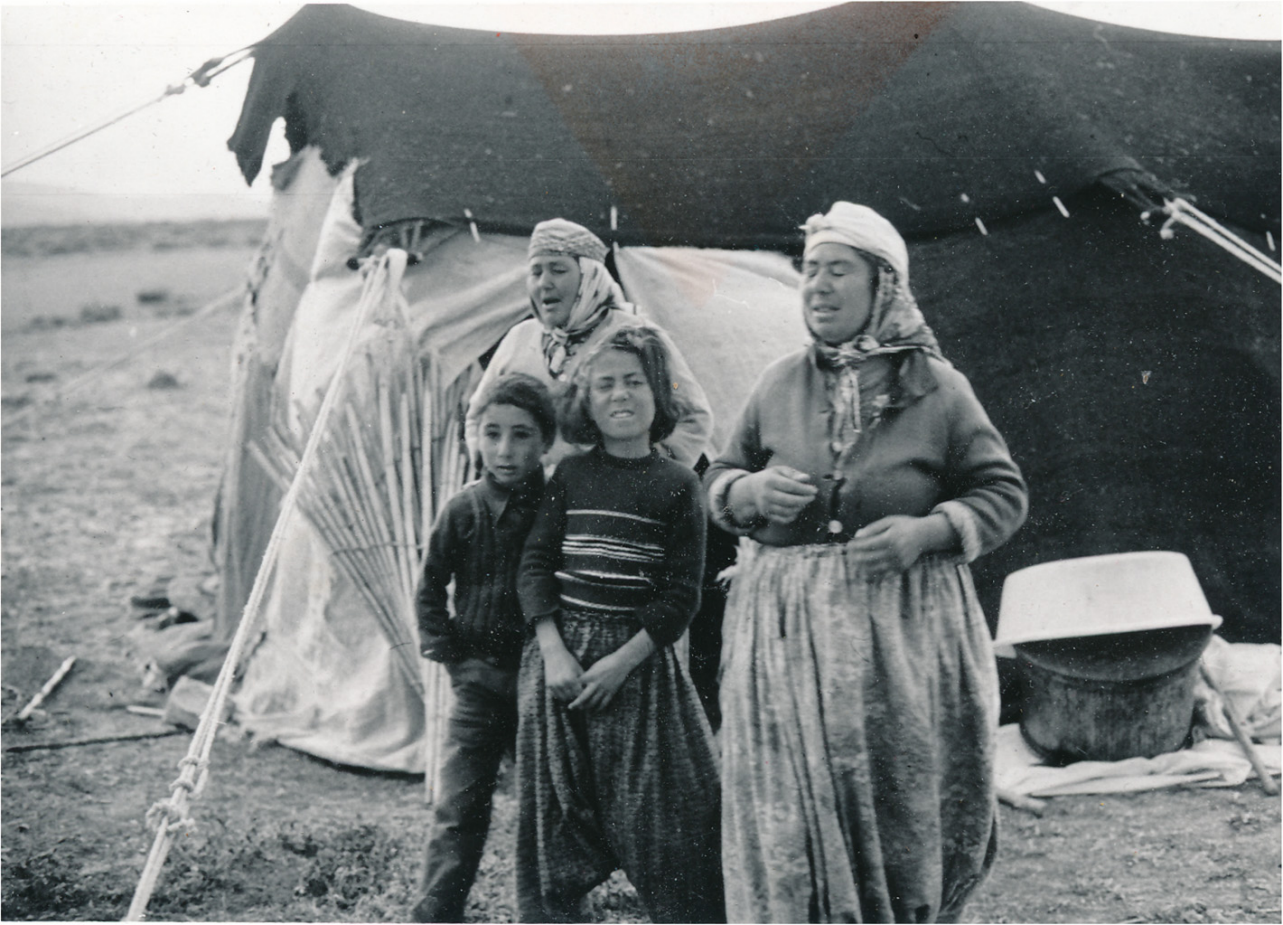
Karahacılı Yörüks in the summer camp outside Niğde (Photo Ingvar Svanberg).

This particular group of Yörüks also had some contacts with itinerant peddlers, craftsmen and horse traders, categorized as *çingeneler* (‘Gypsies’), who regularly set up their tents near the Yörük neighbourhood. Such itinerant groups or service nomads were constantly on the move in Anatolia. They made a living as peddlers, brush-makers, sieve-makers, bear-trainers, or horse-traders, combined with harvesting, cotton-picking, plant gathering, etc. [11]. I used to pay visits to these camps during my stay in Niğde, and I once found in a tent a cage with a rock partridge (*Alectoris chukar*). Thirty years later I used this observation in an essay on the history of aviculture (Figure 2).

Next to the Yörük neighbourhood was a large cement factory which employed townsmen. My intention was to study how the Yörüks occupied their ecological niche as shepherds, their relationships with other ethnic groups in the vicinity, and how they could survive as semi-nomads in a rather industrialised context. Yörüks I had seen elsewhere along the southern Anatolian coast had switched to green house economy, while this group in Niğde continued as shepherds and semi-nomads. This was promising. They kept a traditional lifestyle using locally available resources, literally on the margins of an industrialized society.

I became acquainted with these Yörük families when visiting the town during the summer of 1978 and the spring of 1979. At one of my first visits to them we were shearing sheep wool. My first host, the oldest brother in the quarter which I liked very much, was not prosperous enough to house me for a longer stay, according to the Yörüks. Instead I moved into the house of Ali, another brother in the lineage group of the Karahacılı tribe that constituted the settlement. He said he was willing to house us for a few months and to teach me the Yörük way of life. With his two wives, many children, and a large house, he was the most prominent of the brothers of the lineage group. He had fifteen cows, one very large bull and around two hundred sheep. I managed to record some kinship terms, asked about marriage patterns, and did some elementary surveys of the households. One day the Yörük women were baking, using cattle dung as fuel in the oven. We completed an inventory of the simple material cultures of the homes (they had TV—still not commonplace in Anatolia at that time). I recorded what was possible with my limited experience and shortcomings in the language, and I must admit that everything was very elementary. I had a handwritten black notebook with questionnaires I had compiled. Nothing on wild plants could be gathered at this stage and few data about animals. These Yörüks put the first shed tooth in a piece of bread and gave it to a dog (like Central Asians do)! I also recorded a few notes about the danger of dogs (in folk religion).

Ali soon came to be more of an obstacle than help because of his exuberant hospitality. He prevented me from visiting other households of the Yörük neighbourhood. I was not allowed to participate in the sugar beet harvest or anything else that Ali thought that I, as his guest, should not have to do. I really tried. I went out in the fields, used the hoes and took part in cutting the beets, but was immediately sent back to the house when Ali found out. Of course it was a matter of communication, or, rather, lack of communication. My knowledge in Turkish was still very limited. I had only a few weeks of modest training. To collect 24-hour recall data on food intake or detailed household inventories and surveys was not an option.

Thus, I was placed within the walls of the house, and my host ordered one of his little daughters always to be at hand to attend me. Ali had a big Anatolian watch dog, trained to keep thieves, wolves and other predators away from his sheep, and of course also unwanted guests from his house. Every stranger was a potential thief in this dog’s eyes. To study dog keeping was one of my aims. But this dog made it impossible for me to leave the house, even for small necessary walks, without the company of any of the house’s children. The outhouse was a primitive round stone building in the back-yard. It had a hole in the wooden ground. The building was actually covered with bricks of dried cow dung, used as fuel. On the roof was a big haystack.

**Figure 3:**
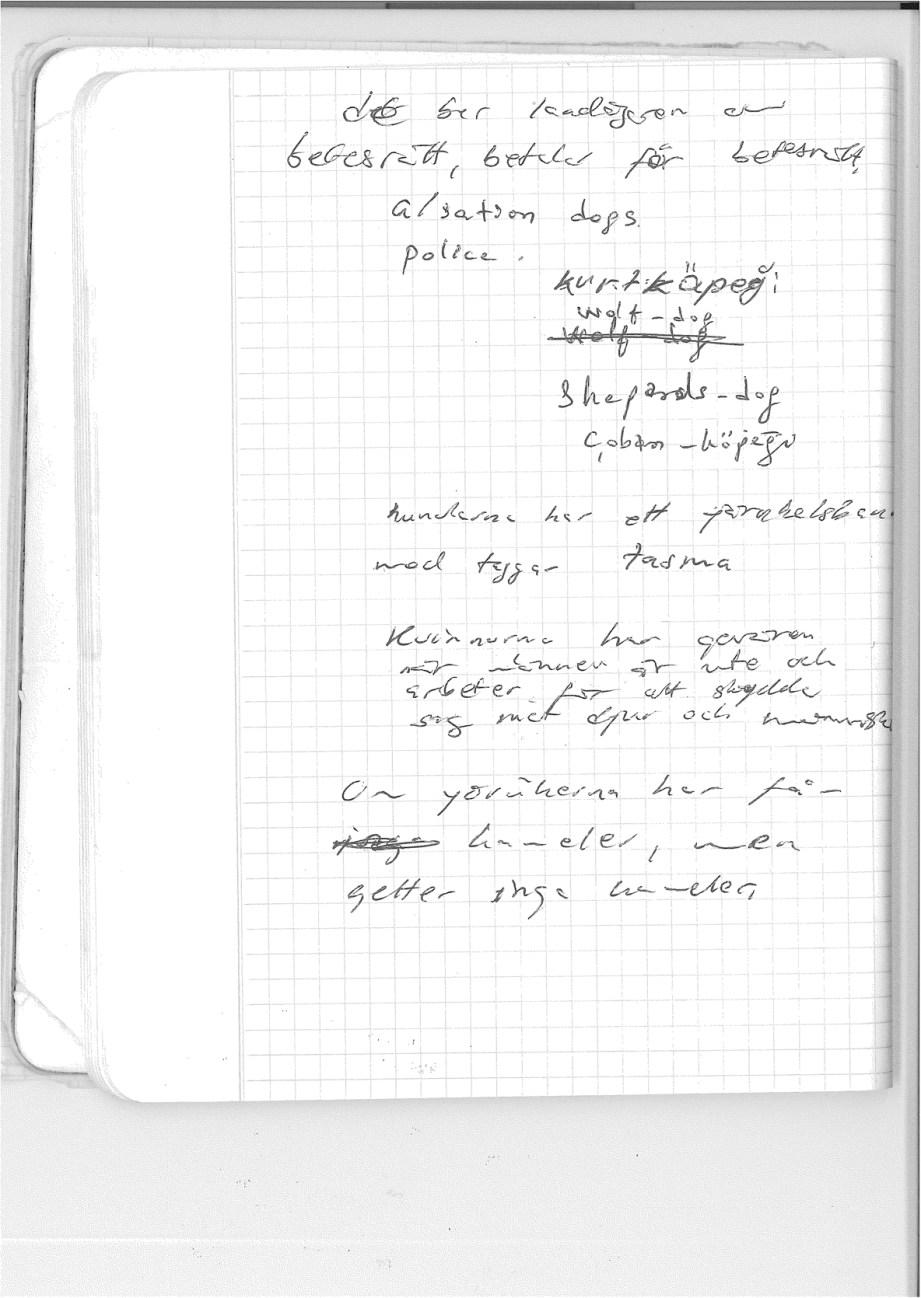
A page from my Anatolian field notebook from 1979 with questions and annotations on dogs.

It might have been possible to endure these enforced restrictions on my freedom and still succeed in doing good field work. I could at least have won the dog’s favour. I am not afraid of dogs, but I certainly respect these Anatolian sheepdogs kept by the Yörüks. Although studying their way of dog keeping was my aim, I was not exactly prepared for the situation. What could I ask, really, about these big, lazy dogs mostly sleeping in the yard and only reacting when someone passed by? (Figure 3).

However, the bites of the myriad bedbugs and fleas in the beautiful carpets, which had the special patterns of the Karahacılı tribe, were rapidly becoming so severe that it would have been impossible to stay confined there for several months more. The infested carpets were our beds. I had no romantic view or illusions about field work. I knew it would be hard, providing many shortcomings when it came to hygienic aspects. We had, for instance, no tap water, and hot water did not exist. But even cultural relativism has its limits. The situation was awkward to me. Participant observation was not the working method here. To change houses, but still continue to visit families in the Yörük neighbourhood was unthinkable as it would have been a direct affront to Ali. The only way out of this was to leave, and try to find another Yörük settlement elsewhere willing to accept me.

## Switching fields

The solution came in the form of a Kazakh refugee from Xinjiang who lived in Niğde. He was aware of my field work and research. I am not sure that Ali ever quite understood why I was living in his house. He was a host showing me Yörük hospitality. This Kazakh, Mr. Qaplan, offered to let me live in his apartment for a while and then visit other refugee families in his home-village Altay köyü out in the Anatolian countryside. I had earlier visited his village in order to record some Kazakh folk music, and I was then fascinated by the hospitable people in this, as I then thought, very isolated village on the Anatolian high table land. Furthermore, I had read articles about the historical background of the Kazakh refugees and also acquired some general knowledge of Kazakh ethnography prior to travelling to Turkey. So even if European ethnology was my field (and Turkey was at least on the margin of Europe), I was not a complete novice to Central Asian cultural history and ethnography. This became a new career move for me. I worked for many years with Kazakh, Xinjiang and Central Asian matters. Geopolitical changes with the breakdown of the Soviet Union kept me studying this geographic area for many years. I also kept an ethnographic and academic interest in Turkey, Turkish migration and Turkish Islam. This was all good for my career as a scholar, but it steered me away from folk biology. Ethnobiological matters were therefore of little importance in these lectures and publications [12].

My field work among the Karahacılı Yörüks in Niğde failed. It did so partly because of the fierce dogs and itchy parasites, but probably more because of my mistaken belief that participant observation was the best method to study the things I was looking for. I never managed to carry out any further research of their interrelationship with the biota, nor did I ever have the opportunity to do studies among the related Juruči of the Plačkovica Mountains in eastern Macedonia. However, these experiences among the Anatolian Yörüks inspired me to successfully study locally known plant repellents against fleas and bedbugs, although in much later, in Estonia [13]. I am often thinking of questions about the Juruči that I never had a chance to pursue, and I have been looking for an opportunity to go back ever since, but so far without success [14].

Although my Yörük field work took a new direction, and I spent many years working with Kazakhs from Xinjiang emphasising macro-perspectives (social structure, impact of ideologies, law systems and politics) rather than micro-perspectives with focus on local environmental knowledge, I continued to keep an eye on folk botany and folk zoology. I believe we developed a still useful theoretical model for researching the use and non-use of locally available food resources in a peasant setting [15,16]. My Kazakh studies kept at least my interest alive. Horses, dogs and hunting eagles were all important key symbols in the masculine Kazakh worldview, which I of course had come to understand. I also returned to historical studies and found a very valuable source―written by Johan Peter Falck, a pupil of Carl Linnaeus―with data on Turkic (Kazakh, Bashkir, Tatar, Siberian Turkic groups) ethnobotany and ethnozoology, of which I published a part in 1987 and have continued to publish since [17,18].

The spirit of the time also turned my interest towards Islam and folk religion among Turkic peoples, aspects which I still find interesting, also from an ethnobiological perspective [19]. Through the Kazakhs I could also study various aspects of folk biology and folk therapy. For instance they told me about a cure against rheumatism: the Kazakhs in Xinjiang used the skin of a one year old fat lamb. The whole skin was put on the body and filled with spices. It was later fascinating to find out that exactly the same folk therapy was used among medieval Mongols [20,21]. I even recorded a story about two hunters capturing a female relative to the abominable snowman in the Altai Mountains in the 1930s, a story that later became a motif for a scholarly paper intended for an anthology on cryptozoology [22].

In the early 1990s, when I returned to Central Anatolia in order to investigate some thirty-five villages in the Kulu district from where many immigrants in Sweden originated, my colleague and I also asked about local plant use. This time I was better prepared. Now we actually recorded some information, but never had the time to do any systematic data collection. *Sıyırma* (*Circium arvense*) served as a distraction for workers in the fields. In April, its juicy marrow was regarded as very delicious, and it was eaten on the spot when they came across it. Also *deve kengeri* ‘camel thistle’ (*Silybum marianum*) was eaten. It was common in earlier times for poor families to harvest thistles and chop them into pieces for a supplement to the animal fodder. *Ayrık* was a small, unidentified plant (probably *Elytrigia repens*), with long roots that were used for making strings. In ditches along the fields grew *sabun otu* ‘soap grass’ (*Saponaria officinalis*), which they earlier used to produce soap with. Very common was the fragrant plant *yavşan* (*Artemisia vulgaris*), which made excellent tinder for lighting the fire. A plant locally known as *bilecik çiçeği* (unidentified) had been used for making rosaries (*tespih*). I am sure the Yörüks in the late 1970s could have provided me with similar information and much more, if we could have had more time together.

Through research among immigrants in Sweden I became more and more interested in contemporary local plant use (e.g. ferns gathered by Koreans for food in Sweden; leaves of common sorrel (*Rumex acetosa*) picked by Turkish women in the spring; mushrooms harvested by Thai immigrants), but I never had the chance to explore this further. (Ethnic Swedes themselves are by the way currently harvesting large quantities of wild berries, such as cloudberry (*Rubus chamaemorus*) and cowberry (*Vaccinium vitis-idaea*) in the forests). I did however contribute later to scientific articles on contemporary wild utility plants in the North and the Baltic Rim [23,24].

## Back in the field

Fate brought me to the Faroe Islands in the mid-1990s, where people then, and now, still rely very much on the marine biota: fishing, whaling (pilot whale), and fowling (northern fulmar, puffin, common guillemot). Richard J. Ford’s 1994 book coincided with my first visit to the Faroes and it inspired me to begin researching folk biology knowledge there [25]. My work since then has led me to the discovery of interesting ethnobiological data [26-30]. My extended stay there (no permission needed, no real language barrier) amidst the rain and mist also gave me time to finish various books I had been planning for a long time. I looked through my files of historical excerpts from Sweden and there was plenty of material for a handbook on traditional plant knowledge. This was later followed by other Swedish language handbooks (fish, mammals, herptiles and birds) within ethnobiology.

I was now firmly back on the ethnobiological track again, trying to convince foundations and grant agencies to provide me research money— sometimes with success, sometimes not. Methodological aspects especially interested me [31,32], as well as the discipline’s history. A brief field study among sheep farmers in Greenland in 2000 convinced me of the need for further field research elsewhere in other parts of the world. Today I am working with other ethnobiologists and colleagues from different fields worldwide. Previously, ethnobiology was very lonely business. Nowadays I cooperate with scholars from Finland, Estonia, Faroe Islands, Germany, Great Britain, Iceland, Italy, Hungary, Lithuania, Poland, Russia, Spain and the United States. I am researching a wide spectrum of interesting topics: local plant knowledge in Eurasia, whales in folk religion, traditional fishing, activity contexts between human beings and animals in circumpolar areas, cultivated plants, history of our discipline, and I am especially interested in the importance of companion animals (dogs, cage birds, wild animals as pets) in various societies. Local aquaculture is another important research field [33-41]. Some of these studies are based on the field material once gathered in Turkic contexts. I am convinced that ethnobiology in European and northern Eurasian environments provides rewarding fields, which need more support and many more scholars [42].

Ethnobiologists study a vast array of knowledge. Academically, ethnobiology is still forming as a discipline and its scope is broad by design. However, as I see it, we examine the biocultural domains that develop through the interactions between human beings and other organisms. It is an active process affecting all parts of any ecosystem. We are therefore interested in a dynamic complex of information and data about the biota we study. Contextual aspects are essential for all ethnobiological research; and I find the emic perspective fundamental. For me, nature *is* culture. List-making, for instance of medicinal plants, might sometimes be useful, but could also be misleading in terms of understanding of the interrelationship between humans and other species. Readings of Claude Lévi-Strauss’ *Le pensée sauvage* (1962), Ian Saep Majnep’s and Ralph ’s, *Birds of My Kalam Country* (1977), Richard Nelson’s *Make Prayers to the Raven: A Koyukon View of the Northern Forest* (1983), William Balée’s *Footprints in the Forest* (1994), together with Ragnar Kinzelbach’s (1999) writing on cultural zoology, have given more theoretical strength to my way of viewing our discipline. Few yet call themselves ethnobiologists [43-47].

How I wish I had read them before I went into the field for the first time! But who knows— if I had, would I be doing what I am doing today? For me it all started with a failure in Anatolia; still it was possible for me to do some research, although for almost two decades I focused more on other aspects of subsistence and survival, including life-styles and world-views. Ethnobiological field work also taught me the virtues of combining various kinds of sources. I read many other scholarly works and maintained other theoretical interests. It was not until my experience in the Faroe Islands that I became more specialized in what we now call ethnobiology.

The Yörüks of Anatolia will remain an interesting topic, but for someone else. I am sure that ethnobiological field investigations of great quality are still possible among them. I strongly encourage younger scholars to go out in the field whenever they have a chance. It is in many ways much easier nowadays. And I can assure you, rich field work material is an investment for many years of research to come. It is very much fun, too.

## Competing interests

The author declares that he has no competing interests.
